# A Proof of Concept Study on Individual Trends in Suicidal Ideation: An Ecological Momentary Assessment Study of 5 Patients Over Three Months

**DOI:** 10.17505/jpor.2023.25265

**Published:** 2023-06-17

**Authors:** Chani Nuij, Wouter van Ballegooijen, Arnout C. Smit, Derek de Beurs, Remco F.P. de Winter, Rory C. O’Connor, Ad Kerkhof, Jan H Smit, Heleen Riper

**Affiliations:** 1 Section Clinical Psychology, Amsterdam Public Health Research Institute, Vrije Universiteit Amsterdam, Netherlands; 2 Department of Psychiatry, Amsterdam Public Health Research Institute, Amsterdam UMC - Location Vrije Universiteit Amsterdam, Netherlands; 3 Mental health institution GGZ Rivierduinen, Netherlands; 4 Suicidal Behaviour Research Laboratory, Institute of Health and Wellbeing, University of Glasgow, UK

**Keywords:** Suicide, ecological momentary assessment, experience sampling methods, time-series analyses, complex systems, early warning signals

## Abstract

**Background:**

Suicidal ideation (SI) is a significant and long-lasting mental health problem, with a third of individuals still experiencing SI after two years. To date, most Ecological Momentary Assessment (EMA) studies of SI have assessed its day-to-day course over one to four consecutive weeks and found no consistent trends in average SI severity over time.

**Aim:**

The current proof of concept study assessed daily fluctuations of SI over a time span of 3 to 6 months to explore whether individual trends in SI severity could be detected, and if so, if the trajectory of changes were gradual or sudden. The secondary aim was to explore whether changes in SI severity could be detected at an early stage.

**Method:**

Five adult outpatients with depression and SI used an EMA app on their smartphone in addition to their regular treatment for 3 to 6 months, where SI was assessed 3 times a day. To detect trends in SI for each patient, three models were tested: a null model, a gradual change model and a sudden change model. To detect changes in SI before a new plateau was reached, Early Warning Signals and Exponentially Weighted Moving Average control charts were used.

**Results:**

In each patient, average SI severity had a unique trajectory of sudden and/or gradual changes. Additionally, in some patients, increases in both sudden and gradual SI could be detected at an early stage.

**Conclusions:**

The study presents a first indication of unique individual trends in SI severity over a 3 to 6 months period. Though replication in a larger sample is needed to test how well results generalize, a first proof-of-concept is provided that both sudden and gradual changes in SI severity may be detectable at an early stage using the dynamics of time-series data.

## Introduction

Suicidal ideation (SI), defined as thoughts of intentionally ending one’s life (O’Connor & Nock, 2014), is a significant health problem. The estimated lifetime prevalence of SI is almost 10% of the world’s population (Borges et al., 2010; Nock et al., 2008). Longitudinal cohort studies indicate that, in general, SI severity decrease over time (Borges et al., 2008; Have et al., 2009; Kivelä et al., 2019). However, a third of individuals still experienced SI after two years (Have et al., 2009; Kivelä et al., 2019), four years (Kivelä et al., 2019), and even after ten years (Borges et al., 2008), thus demonstrating the long-term course of SI.

It has been argued that the course of SI should be studied in the context of dynamical systems theory (Beurs et al., 2021). This theory describes the complex behaviour of systems and, according to this theory complex systems can have different states within an individual (Cramer et al., 2016; van de Leemput et al., 2014; Wichers et al., 2020). Evidence of different states of SI within individuals (e.g., more severe or less severe SI) was found in a military sample with acute suicide risk (Bryan & Rudd, 2018). This latter study made use of a weekly tracking log that assessed the severity of SI.

To gain more insight into the daily fluctuations of SI, recent studies have applied ecological momentary assessment methods (EMA; Stone & Shiffman, 1994). EMA methods use repeated measures, for example throughout the day for multiple consecutive days, in order to capture the finergrained variation of symptoms from day-to-day (Myin-Germeys & Kuppens, 2022). A systematic review of 35 EMA studies of SI found that the intensity of SI can fluctuate in a matter of hours, SI can have a high variability and there are large individual differences (Sedano-Capdevila et al., 2021). So far, most EMA studies analysed daily fluctuations of SI during one to four consecutive weeks (Sedano-Capdevila et al., 2021) and found no significant changes in average SI severity over time (Kleiman et al., 2017; Rath et al., 2019), meaning that there seems to be no discernible trend in the daily fluctuations in SI when you assess its course within a month. It is possible that a month is too short a time frame to detect fluctuation patterns. Indeed, studies from the field of depression have found individual trends in depressive symptoms when looking at a time period of three to six months (Smit, Snippe, et al., 2022; Wichers et al., 2020).

Individual trends put daily fluctuations in context, for example, by estimating whether a sudden spike of symptom severity is part of an overall increase in symptom severity or is more appropriately viewed as an isolated outlier. A change in a trend can be gradual, meaning that the average level of severity slowly increases or decreases over time, or sudden, which represents a sudden persistent increase or decrease in symptom severity. It would be meaningful to detect a change in a trend at an early stage, meaning before the symptom severity reaches a new plateau, making personalized risk assessment and intervention possible. In depression research, assessing these so called Early Warning Signals (EWS) have received a lot of attention in the past decade, showing that changes in a trend at an early stage are somewhat more common before transitions towards higher (Curtiss et al., 2021; Olthof et al., 2020; Smit, Helmich, et al., 2022; van de Leemput et al., 2014; Wichers et al., 2020; Wichers & Groot, 2016) and lower levels of depression (Helmich et al., 2022; Smit, Helmich, et al., 2022).

In the current proof of concept study, we assessed the daily fluctuations in SI over a time span of 3 to 6 months. Our primary aim was to explore whether a trend could be detected in SI severity for each individual participant, and if so, if the trajectory of changes was gradual or sudden. The secondary aim was to explore whether changes in SI severity can be detected at an early stage.

## Method

### Design

This study consists of 5 single-case time series studies, in which depressed patients at risk of suicide rated their SI three times a day. Data from this study were collected as part of the CASPAR study (Nuij et al., 2022), which investigated the feasibility of using mobile EMA and mobile safety planning as components of the regular treatment of depressed patients with suicide risk. In this feasibility study, patients were asked to use a mobile EMA app for at least 3 months, and up to 6 months on a voluntary basis as part of their treatment.

### Participants

Participants were recruited among adult outpatients or day-care patients in specialised mental health care in the Netherlands. They were included when, based on the clinical view of their clinician, they had depressive disorder, SI and no psychotic symptoms. Additionally, they had to be proficient in Dutch and in possession of a smartphone (either Android or iOS). Participants were recruited between March 2019 and March 2020. This yielded a total sample of 17 participants (Nuij et al., 2022). For the current study, we wanted to investigate trends in SI for 3 months or more. Therefore, we included in our analysis patients who had enough data to analyse: on average 2 or more observations a day during at least 90 days; 180+ observations for each patient. Five participants met this minimal data requirement. Participant characteristics are shown in table 1. These 5 participants were similar to the original 17 in terms of age, employment, marital status and clinical characteristics, but differ in gender distribution. While the original sample consisted of 9 males and 8 females, 1 male and 4 females had sufficient observations.

**Table 1:** Patient characteristics and number of SI observations

Patient	Gender	Lifetime suicide attempt	Age range	*N* observations of SI
1	F	0	40-50	186
2	M	0	20-30	282
3	F	0	40-50	195
4	F	0	20-30	180
5	F	1	20-30	187

SI = suicidal ideation; F = female; M = male

### Procedure

Patients were invited to participate by their clinician. Those who consented to participate signed the informed consent form and completed baseline assessments face-to-face at the outpatient clinic. Patients answered online questionnaires, including the Self-Injurious Thoughts and Behaviours Interview (SITBI) (Nock et al., 2007). Afterwards, the researcher helped the patient to install the EMA app on their smartphone and explained how to use the app. The contents of the EMA items were also explained in detail. After the meeting, patients could immediately use the app independently. The EMA app was used in addition to their regular treatment. Five telephone interviews per patient were conducted, which included a question on life events. In case of a sudden change in suicidal ideation, we inspected whether this coincided with life event reported by the participant.

The study lasted 3 months, but patients and their clinicians could decide to use the EMA app for another 3 months if they jointly decided it would have benefit. Patients received incentives by means of a discount code for a large online retailer, partly depending on a completion of over 60% of the EMA prompts in the first month after inclusion, with a maximum of 20 Euro.

#### EMA procedure

The smartphone app mEMA (mobile ecological momentary assessment; Ilumivu.com) was used for the data collection. This is a smartphone-based EMA program that runs on both Android and iOS smartphones. A semi-random EMA design was employed with a sampling rate of three random prompts between 9:30 AM and 6 PM. This survey contained 12 to 14 items (follow-up questions were asked based on given answers).

In the current study, we used one EMA item that assessed SI. Patients were asked to rate the item ‘I have the desire to end my life’, rated on a 7-point Likert-type scale with the answer options: 1 (completely disagree), 2 (disagree), 3 (somewhat disagree), 4 (not disagree nor agree), 5 (somewhat agree), 6 (agree) and 7 (completely agree). Therefore, answers of 1 or 2 indicate no suicidal thoughts, answers of 3 or 4 some suicidal thoughts, and answers of 5 to 7 are a confirmation of suicidal thoughts ranging in intensity. To check for life events, we inspected answers to an open EMA item: Did something happen today that influenced you, or do you have other remarks.

### Analytic Strategy

#### Trend analysis

To detect trends in the SI data for each patient, we tested three models. We tested a null model that assumed no trend (a constant rolling mean, i.e., a flat line), a gradual change model that assumed smooth transitions, and a sudden change model that assumed sudden transitions.

To model gradual change, a local linear model (LLM) was fitted to the SI time series of each of the 5 patients using the ‘np’ package in R (Racine & Hayfield, 2020; R Core Team, 2019). The LLM fits a weighted linear regression to the data at each of the data points Yt, where data points closest in time to Yt receive the highest weight. The LLM uses a global bandwidth parameter, that determines the rate at which weights around Yt will decay, thereby controlling how smooth or wiggly the fit is. Package ‘np’ can also estimate the 95%-CI for the slope estimated at each point Yt. This was used to test where the slope is significantly different from 0, estimating the significance of gradual trends.

To model sudden change, a change point model (CPM) was fitted using the ‘strucchange’ package in R (Zeileis et al., 2015). In the CPM, the data are assumed to be distributed around a stable mean, and the CPM aims to identify if and when the trend changes from one stable mean to the next. The CPM divides the trend in segments. Within each segment, the data are assumed to be distributed around the same mean. Between these segments, the trend changes abruptly from one mean to the next at so-called change points.

The LLM, CPM, and a null model that assumed no trend were compared with each other using Leave-One-Out Cross-Validation (LOOCV; Lachenbruch & Mickey, 1968). In LOOCV, the model is fitted using all but one of the observations, the remaining observation is then predicted based on the fit obtained. This procedure is repeated so all observations are left out once. The closer predictions are to the real data, the better the model fits. By comparing these models using LOOCV, we could quantify whether the course of SI can be considered to be (a) part of a smooth curve (LLM), or (b) part of a distinct phase with a constant mean (CPM). When the null model performed best, we concluded that no long-term trend was present. It is possible that the trend in SI can best be modelled using a combination of gradual and sudden changes. To assess this, plots of the raw data and fitted trends were inspected visually.

In some cases, data-driven estimates may lead to small bandwidths for the LLM or small segment sizes for the CPM, and models may end up fitting primarily short-term fluctuations, rather than the medium-term trends that are the focus of this study. To avoid this, the minimum bandwidth and segment length were set to 2 weeks.

#### Detecting changes in trends at an early stage

To detect changes in SI at an early stage, additional analyses were performed. For gradual changes, we used Exponentially Weighted Moving Average (EWMA) control charts to test if rises in the level of SI could be detected in real-time. This technique indicates that a gradual change is in progress if the average level of an individual’s severity changes significantly (e.g., compared with an earlier time frame), assuming that a sudden change has not occurred. A weighted average of recent observations in SI was calculated after each observation using: EWMAt = λ × Yt + (1 – λ) × EWMAt–1. After each observation, the estimated EWMA was compared with a bandwidth calculated based on a run-in period of 63 observations (i.e., the number of beeps in 3 weeks). If the EWMA exceeded the bandwidth, the level of SI is significantly higher than in the run-in period, which can be seen as a real-time indication SI is worsening. Model choices were based on Smit et al. (2019), which means (a) outliers were Winsorized, (b) we controlled for autocorrelation if the auto-regressive(1) model had a lower corrected Akaike Information Criterion (AICc) than the auto-regressive(0) model, and (c) control parameters were set to λ=0.01 and L=3. See Smit, Schat & Ceulemans (2022) for technical details.

For sudden changes, we first inspected the participant’s comments to see whether the change followed directly on a life event. If not, we investigated if sudden changes were preceded by two commonly used EWS: increases in autocorrelation and variance. To see the changing pattern of auto-correlation and variance over time, moving window techniques were applied using time-windows of 63 observations (i.e., the number of beeps in 3 weeks). For each observation from the 63^rd^ on, autocorrelation and variance were estimated using data from the previous 63 observations. To test if autocorrelation and variance were rising when closer to the transition, as hypothesised, the Kendall rank correlation was calculated between the window estimates and their time index.

To estimate the autocorrelation in non-equidistant data, the nls() function in R was used to solve for a in the following function: Y_t_ = Y_t-1_ × e^aΔt^, where Δt is the time in hours between Y_t-1_ and Y_t_. This will then be converted to a 5.5-hour autocorrelation using: autocorrelation = e^a × 5.5^. Note that, though 5.5 hours was chosen to match the interval between observations in Wichers et al. (2020), any interval would lead to the same results.

## Results

### Participants Characteristics

Participants were four females and one male. Four participants had a depressive disorder and one participant dysthymia. All participants reported experiencing current SI on the SITBI, which was confirmed by their clinicians. One participant had attempted suicide in the year before enrolment in the study, other participants reported no lifetime suicide attempts. Participants completed an average of 206 EMA surveys on SI (*SD* = 42.82), yielding a total of 1030 EMA assessments (see Table 1). Participants answered on average 1.84 assessments per day (out of three prompts), resulting in a compliance rate of 61.5%.

### Trend Analysis

**Figure 1:**
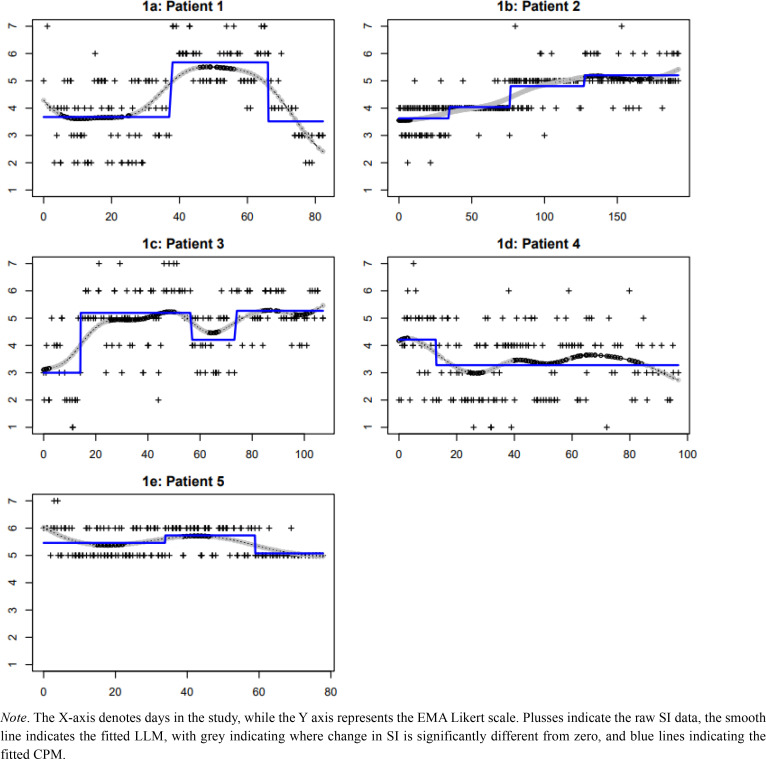
Plot of SI EMA data for each participant with estimated long-term trends.

**Figure 2:**
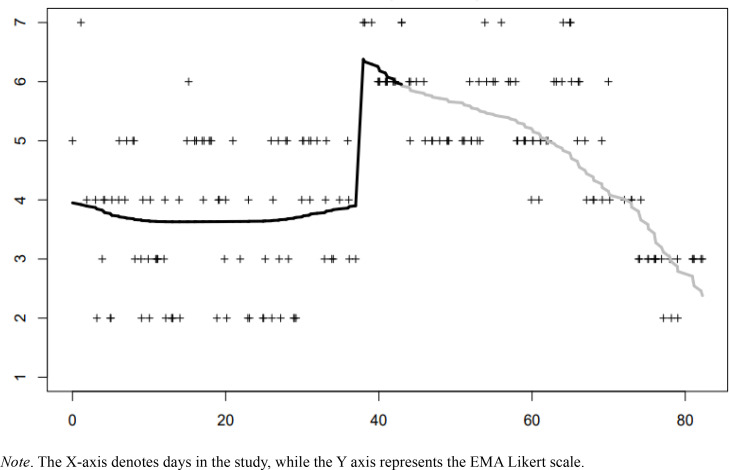
Sudden-gradual regression of patient 1.

Figure 1 shows the individual plots of SI with the estimated long-term trends, and Table 2 shows the results of the LOOCV. Based on visual inspection, it seems that patient 1 experienced both types of change during the research period: SI increased suddenly from an average of almost 4 (neither disagree nor agree) to an average of almost 6 (agree), and then decreased gradually towards having less intense suicidal thoughts at the end of the research period (Figure 1a). Post-hoc re-analysis with a statistical method capable of fitting both sudden and gradual changes in the same time series (Smit, 2020) confirms our visual inspection that the increase was sudden, whilst the decrease was gradual (see Figure 2). As LOOCV cannot be obtained for this method yet, we cannot confirm numerically whether the sudden-gradual regression fit the data better than the LLM and CPM.

**Table 2:** Comparison of trend models using Leave-One-Out Cross-Validation

Patient	Local linear model	Change point model	Null model (no change)
1	**0.881**	0.896	*1.213*
2	**0.409**	0.415	*0.676*
3	0.843	**0.808**	*0.975*
4	**1.047**	1.101	*1.112*
5	0.407	**0.405**	*0.510*

*Note*. **Bold** numbers indicate the best fitting model for each patient, *cursive* numbers indicate the worst fitting model. Lower numbers indicates better fit.

In patient 2, both LOOCV and visual inspection seem to indicate the trend in SI started with a long gradual upward trend from an average of almost 4 (some suicidal thoughts) to a seemingly stable period with an average slightly above 5 (suicidal thoughts with low intensity) (Figure 1c). In patient 3, both LOOCV and visual inspection seem to indicate that SI increased suddenly in the first month of the research period from some suicidal thoughts, followed by a relatively stable period with some fluctuations in intensity of her suicidal thoughts (Figure 1d). In patient 4, a small decrease in SI can be seen in SI from a score of 4 to 3 (some suicidal thoughts) (Figure 1e). In patient 5 we see fluctuations between scores 6 and 5, indicating the intensity of suicidal thoughts (Figure 1f).

### Detecting changes in trends at an early stage

Average SI severity of patients 1, 2, and 3, increased 2 points on the 7-point scale during the research period, meaning a notable increase in SI, and we explored whether these changes could be detected at an early stage.

**Figure 3:**
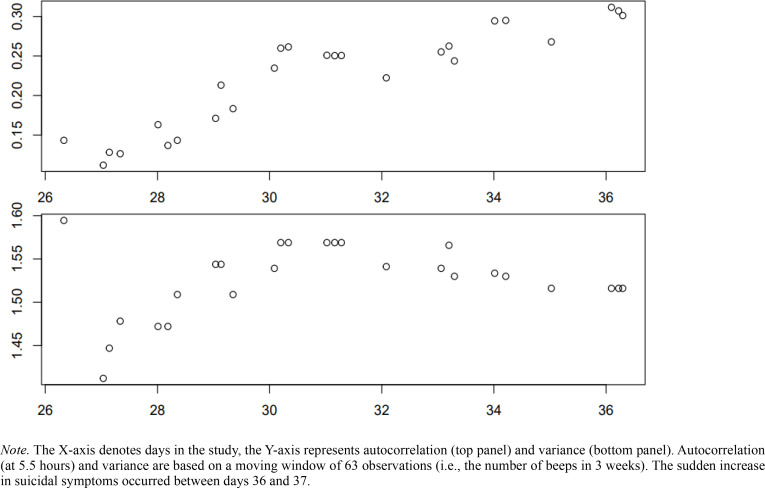
Trend in estimated autocorrelation and variance of SI of patient 1 preceding the sudden increase in SI.

In patient 1, a sudden increase in SI was found. After inspection of interview data and the open EMA item, we concluded that the sudden increase did not follow a specific event. We investigated if this increase was preceded by EWS like an increase in autocorrelation and variance. Figure 3 shows the trend in estimated autocorrelation (top plot) and variance (bottom plot) over time. Whilst the autocorrelation increased significantly before the transition (Kendall *τ* = .778, *p* < .001), the variance did not (Kendall *τ* = .068, *p* = .640).

**Figure 4:**
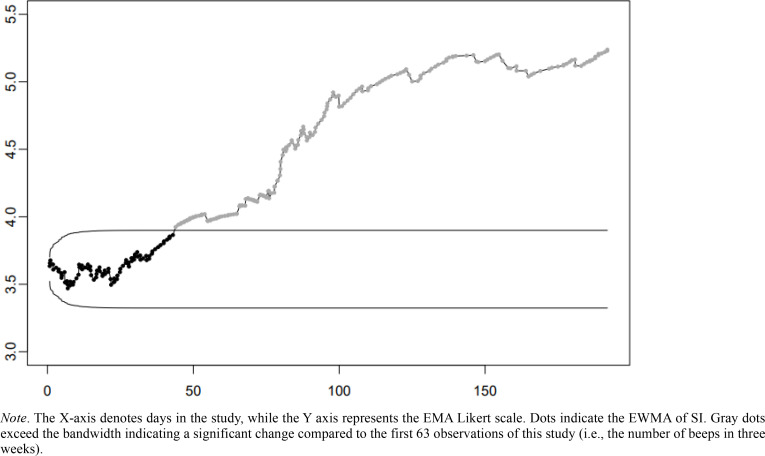
Exponentially weighted moving average control chart of SI severity of patient 2

In patient 2, a gradual increase in SI was found. EWMA was used to test if rises in the level of SI could be detected in real-time at an early stage. Figure 4 shows that for this patient, the level of SI exceeded the bandwidth on day 43 of the study, more than two months before SI appeared to reach a new plateau around day 100 of the study.

Though patient 3 also showed a sudden increase in SI, this increase occurred at observation 56. This means that no windows of 63 observations could be constructed before this increase and it was impossible to investigate EWS in this participant.

## Discussion

This EMA study with 5 outpatients showed that momentary levels of SI severity fluctuated around longer-term trends. In each patient, average SI severity had a unique trajectory of sudden and/or gradual changes across three to six months. Additionally, we found that a sudden increase in SI severity in one patient was preceded by slower hourly fluctuations in severity (rising autocorrelation but no change in variance). In another patient, a gradual rise in SI could be observed at an early stage. These findings provide a proof of concept that trends in suicidal ideation can be detected over 3 to 6 months. In addition, such findings can inform new methods for prediction of increases in suicidal ideation severity.

Previous studies investigated long-term and short-term changes in SI severity. Cohort studies described that, on the average group level, SI severity tends to decrease over a period of years, but SI was still experienced by one third of patients (Borges et al., 2008; Have et al., 2009; Kivelä et al., 2019). Previous EMA studies found fluctuations in SI severity in a matter of hours and large individual differences (Sedano-Capdevila et al., 2021). Our study indicates that it is possible to interpret momentary changes in the context of a longer-term trend and it sheds some light on how an individual’s average SI severity can change: shift suddenly or gradually change over several weeks or even months. It can be suggested that SI is not stable within a day, week, month (Sedano-Capdevila et al., 2021) or even several months; therefore it is important to continue to monitor patients with SI.

Results showed that most of our patients did not seem to have a single stable state of SI severity. In the context of dynamical systems theory, SI can be hypothesised to have multiple possible stable states within individuals. Two patients in our sample experienced a persistent increase in SI severity and had enough data before the increase to analyse the dynamics of their time-series data. The sudden increase in SI severity of patient 1 was preceded by a significantly slower return of SI to its mean (i.e., rising autocorrelation), but not by increasingly extreme fluctuations in SI severity (i.e., increasing variance), The gradual change seen in patient 2 could be detected in real-time using an EWMA control chart more than 2 months before it seemed to reach a new plateau. Though the application of both EWS and EWMA control charts in EMA data has received a lot of attention recently (EWS: Bos et al., 2022; Helmich et al., 2022; Van De Leemput et al., 2014; Wichers et al., 2020; Wichers & Groot, 2016; EWMA: Schat et al., 2021; Smit et al., 2019, 2022; Smit & Snippe, 2022), these methods had never been studied before in SI data. The current study provides the first empirical evidence that changes in SI severity might be detectable at an early stage before a new plateau in SI severity is reached, based on EWS and EWMA control charts calculated from EMA data.

The strength of this study is the relatively large number of observations for each individual, which allowed single-case time-series analysis. Such data have not been analysed yet in the field of suicide prevention (Sedano-Capdevila et al., 2021). Our assessment schedule of 3 assessments a day during three or more months was able to capture daily fluctuations in SI as well as trends over weeks and months. The feasibility of this assessment schedule is discussed in a different publication (Nuij et al., 2022). However, some limitations must be taken into account. Only a few individuals of the recruited sample could be included in this study due to the large amount of data needed for analysis of an individual. Our study should therefore be considered a proof of concept study. Other trends or no trends might be found in other individuals. Far more participants would be needed to generalize our results to any population. Additionally, the finding that a sudden change in SI severity was preceded by EWS and a gradual change was detected using an EWMA control chart does not imply that these changes in EMA dynamics always indicate upcoming change. Sensitivity and specificity of EWS and EWMA control charts should be investigated in a study that includes many individuals that are at-risk of symptom severity change.

## Action editor

Lars-Gunnar Lundh served as action editor for this article.

## Open access

This article is distributed under the terms of the Creative Commons Attribution 4.0 International License (http://creativecommons.org/licenses/by/4.0/), which permits unrestricted use, distribution, and reproduction in any medium, provided you give appropriate credit to the original author(s) and the source, provide a link to the Creative Commons license, and indicate if changes were made.
